# Associations between Perceptions of Drinking Water Service Delivery and Measured Drinking Water Quality in Rural Alabama

**DOI:** 10.3390/ijerph110707376

**Published:** 2014-07-18

**Authors:** Jessica C. Wedgworth, Joe Brown, Pauline Johnson, Julie B. Olson, Mark Elliott, Rick Forehand, Christine E. Stauber

**Affiliations:** 1 Department of Biological Sciences, University of Alabama, 300 Hackberry Lane, Tuscaloosa, AL 35487, USA; E-Mails: jjcook@crimson.ua.edu (J.C.W.); jolson@as.ua.edu (J.B.O.); 2 School of Civil and Environmental Engineering, Georgia Institute of Technology, 311 Ferst Drive, Atlanta, GA 30332, USA; E-Mail: joe.brown@ce.gatech.edu; 3 Department of Civil and Environmental Engineering, University of Alabama, 245 7th Avenue, Tuscaloosa, AL 35487, USA; E-Mails: pdjohnson@ua.edu (P.J.); melliott@eng.ua.edu (M.E.); 4 Barge Waggoner Sumner and Cannon Inc., 2047 West Main Street, Suite 1, Dothan, AL 36301, USA; E-Mail: rick.forehand@bwsc.net; 5 Division of Environmental Health, School of Public Health, Georgia State University, P.O. Box 3995, Atlanta, GA 30302, USA

**Keywords:** small water supply, rural, water quality, perceived service, drinking water quality, infrastructure, environmental health

## Abstract

Although small, rural water supplies may present elevated microbial risks to consumers in some settings, characterizing exposures through representative point-of-consumption sampling is logistically challenging. In order to evaluate the usefulness of consumer self-reported data in predicting measured water quality and risk factors for contamination, we compared matched consumer interview data with point-of-survey, household water quality and pressure data for 910 households served by 14 small water systems in rural Alabama. Participating households completed one survey that included detailed feedback on two key areas of water service conditions: delivery conditions (intermittent service and low water pressure) and general aesthetic characteristics (taste, odor and color), providing five condition values. Microbial water samples were taken at the point-of-use (from kitchen faucets) and as-delivered from the distribution network (from outside flame-sterilized taps, if available), where pressure was also measured. Water samples were analyzed for free and total chlorine, pH, turbidity, and presence of total coliforms and *Escherichia coli*. Of the 910 households surveyed, 35% of participants reported experiencing low water pressure, 15% reported intermittent service, and almost 20% reported aesthetic problems (taste, odor or color). Consumer-reported low pressure was associated with lower gauge-measured pressure at taps. While total coliforms (TC) were detected in 17% of outside tap samples and 12% of samples from kitchen faucets, no reported water service conditions or aesthetic characteristics were associated with presence of TC. We conclude that consumer-reported data were of limited utility in predicting potential microbial risks associated with small water supplies in this setting, although consumer feedback on low pressure—a risk factor for contamination—may be relatively reliable and therefore useful in future monitoring efforts.

## 1. Introduction

Small, rural, and economically disadvantaged communities in the USA face a variety of challenges, including aging infrastructure. Water supply infrastructure, operation, and maintenance may be sub-optimal in such settings, where revenues for critical investments and ongoing maintenance may be lacking due to such factors as low population densities, geographically large service areas, and limitations on revenues due to an inadequate economic base or rate structures that do not account for the full economic costs of the systems [1]. Although over 80% of the public water distribution systems in the USA are classified as “small” (*i.e.,* serving fewer than 3300 persons), relatively little research characterizing the environmental health risks associated with small water systems in resource-limited settings of the USA has been conducted. Small water systems serve sizeable percentages of the populations of some states (e.g., 25% in Alabama, 58% in Mississippi) and an estimated 58.5 million people in the southeastern USA [2], but they serve a small percentage of the overall US population (~8%). However, small systems have been linked to a disproportionate number of disease outbreaks and have more reported violations under the Safe Drinking Water Act [3,4]. It has also been reported that small systems may experience more frequent interruptions in service, therefore increasing the risk for contamination [5]. From 1997-2012, small water systems in Alabama reported 129 health-based violations per 100,000 people, which is more than 40 times greater than the total number of violations per 100,000 people reported for large water systems during the same reporting period [6]. Thus, in terms of microbial risks, small water systems deserve increased and sustained attention [7].

Despite this need, monitoring microbial quality in small water systems is a challenge. For smaller systems, monitoring resources are modest; therefore their allocation is limited to meeting the regulatory requirements (typically, three samples per month for total coliforms under routine monitoring for systems serving ≤ 3300 people). Gathering reliable, representative data on water quality in rural areas can be logistically complex, costly, and time-consuming, though such data may be useful in identifying priorities for water quality risk management. These data are also required for studies of environmental health that attempt to quantify the association between microbial water quality and health outcomes in populations [8,9].

Self-reported data from consumers is sometimes used to assess drinking water risks, although the predictive utility of such data is not known and may be highly context-specific [10,11]. Evidence from some settings suggests that consumer perceptions of water quality are uncorrelated with measured microbial water quality [12,13]. In this study, we compared matched consumer interview data with point-of-survey, household water quality and pressure data. Our objective was to determine whether consumer interview data and subjective consumer perceptions were predictive of objectively measurable water quality data across a cross-section of 910 households in three counties served by 14 rural water systems.

The counties are typical of Alabama’s “Black Belt”, a historically underserved region whose population faces persistent economic, environmental, and health challenges [14,15]. A majority of the public water systems serving this area are groundwater systems, and all of the systems are treated by chlorination (groundwater systems) or conventional rapid sand filters with chlorination (surface water systems). Of the 14 systems included in the study, six are categorized as “very small” or “small” systems, and eight are classified as “medium” or “large”. In this region, the only alternative drinking water sources are private wells and bottled water. However, wells can be expensive to dig and maintain. Also, it is believed that wells in the region are at risk for contamination from nearby septic systems due to soil and geological conditions [16]. A 2011 study reported that over 50% of the land within the Black Belt was unsuitable for conventional septic systems [17]. Bottled water is expensive and the socio-economic status of the population likely limits their ability to use bottled water as a primary source of drinking water. These characteristics severely limited the options for drinking water that were available to the population. 

## 2. Experimental Approach

As part of a study on water and health in rural water systems in Alabama, a total of 910 households were recruited from three rural counties in Alabama. Prior to household recruitment, Institutional Review Board approval was obtained from the University of Alabama (Approval No.: IRB #10-OR-390-R2). Households were randomly selected from a master list of all households from local utility records. Households that relied on private well water as their drinking water source were not eligible for participation. From an initial list of all eligible households compiled from utility records, we partitioned households into groups of ten, which were numbered and selected for household visits in random order. From January to December of 2012, potential households were approached in this cluster-randomized order and visited by personnel from the study team. When available, the self-designated head of the household was informed about the study and asked if he/she was willing to participate. If the head of household was not available, another member of the household who was ≥18 years of age was asked to participate. Once the household member consented to join the study, an interview was conducted and water samples were taken. 

### 2.1. Household Interviews 

The survey instrument, developed and modified from previous research in the area, consisted of the following sections: socio-economic status, perceptions of water service delivery and aesthetics, access to sanitation services and self-reported gastrointestinal illness [16] (see Supplementary). For water service conditions and aesthetics, the survey asked, for example, “Have you ever experienced intermittent service?”. The answer choices were “Yes” or “No”. If the respondent answered “Yes”, we asked “How Often?” The answer choices were “Less than once in six months”, “At least once per month”, “Daily”, or “Don’t know/no response.” There were no time constraints on the response. Because a previous study in the region did not find associations between the intensity of the water condition and water contamination [16], this survey examined only overall perceptions of water service delivery and conditions. The survey was delivered in-person by a trained researcher, and the oral interview answers were recorded on paper. The surveys were conducted in English at or inside the participant’s home. Only a portion of the data collected in the survey will be addressed in this paper.

### 2.2. Water Samples 

During the interviews, a second trained staff person performed water testing, water sample collection and pressure readings. Microbial water samples were taken at the kitchen faucet to obtain “point-of-use” water quality data and (if available) at a flame-sterilized outside faucet for “as delivered” data. At both sample locations (inside and outside), 100 mL water samples were taken in sterile 120 mL vessels with sodium thiosulfate (IDEXX Laboratories, Westbrook, ME, USA). If possible, we removed any aerator, strainer, or hose that was present prior to sampling. At the outside faucets, the tap was heat (flame) sterilized prior to turning on the flow to ensure that potential microbial contamination on the faucet itself would not affect the water samples. Heat sterilization involved running a small propane blowtorch back and forth on the spigot for approximately 10 s. The objective was to warm the spigot sufficiently to kill any bacteria. The spigot was allowed to cool for 3 min before proceeding [18]. At each faucet, the tap was turned on and flushed for 4–5 min to let the temperature and flow stabilize. Once sampling had been initiated, water flow was not changed to avoid dislodging microbial growth within the faucets or pipes. Each vessel was aseptically filled to the 100 mL mark, closed, and immediately placed on ice for delivery to the laboratory for processing [19]. 

In addition, pressure was measured with two conforming (±5%) Rain Bird pressure gauges (Model P2A, Azusa, CA, USA) on a T configuration (calibrated monthly). No pressure readings were taken from inside households, but microbial and all other measures used point-of-use water samples from the household kitchen. At the kitchen faucet, turbidity (Hach 2100Q Portable Turbidimeter, Loveland, CO, USA), free and total chlorine, and pH were measured (Hach Dual Pocket Colorimeter II plus pH). The 100 mL water samples were processed within six hours of collection for total coliforms and *E. coli* with IDEXX Colilert^®^ QuantiTrays^®^ (IDEXX Laboratories) following the manufacturer’s instructions. QuantiTrays were incubated at 35°C ± 0.5°C for 24 h. After incubation, the positive wells were counted and a Most Probable Number (MPN) was obtained using the included MPN table. 

### 2.3. Data Analysis

All survey and water quality data were entered into a Microsoft Access database, transferred to STATA 10 (College Station, TX, USA) and analyzed. One of the goals of the survey was to determine how customers of small drinking water systems perceived the water service delivered to their households. The survey focused on two key areas of water service conditions: delivery (intermittent service and low water pressure) and aesthetic characteristics (taste, odor and color), providing five condition values per household. To determine whether or not reported problems surrounding water service delivery were associated with measured water quality, we examined the five reported water service conditions (and their frequency) for associations with the six water quality measures. Participants were asked whether or not they experienced any of the aforementioned conditions. If they responded affirmatively, they were asked to report the frequency of that water service condition. Frequency of the experienced condition was categorized into three groups: those never reporting the condition, those reporting it at least once and those reporting the condition at least once per month (monthly) for simple associations. For univariable and multivariable models, the aesthetic and service delivery conditions were dichotomized into “ever experienced the condition” or “never experienced the condition”. 

The following statistical analyses were performed:

(a) Tests for normality on all continuous water quality measures were performed using the Shapiro Wilk test. In addition, distributions were assessed with plots and Tukey outlier detection. Distributions that were skewed were analyzed using ordinal values as model outcomes.
(b) To compare measured water quality across groups of reported water service delivery and aesthetic conditions we used the Kruskal-Wallis tests for continuous variables and chi-square tests for binary outcomes.
(c) For univariable and multivariable regression, we performed the following:
  - For water quality measures that were not normally distributed and federal or state guidelines were available, we transformed the variables into binary outcomes based on suggested regulatory guidelines. Five water quality variables were dichotomized: presence of total coliforms (kitchen and outside faucet samples), presence of free chlorine, presence of total chlorine and turbidity (>0.3 NTU). For pressure, we log-transformed the measure and generated quintiles to produce an ordinal variable.
  - We then performed univariable logistic regression with each reported water service delivery or aesthetic condition as a binary exposure variable and each water quality measure as the binary outcome. For pressure quintiles, we performed ordinal logistic regression with each binary exposure as a predictor.
  - Multivariable models were produced for each of the six water quality outcomes (binary or ordinal) which included each binary exposure and three potential confounding variables (access to sewer, presence of college graduates in the household, and categorical race of household members). Each model was assessed for two-way interactions. Confounding was evaluated by measuring a 10% change in effect size of the odds ratio of the binary exposure in the adjusted model (as compared with the univariable model).

## 3. Results 

This study included 910 households from three rural counties in Alabama (Table 1). The majority of the households were headed by males (54.1%), consisted of adults only (90.9%), and identified as African American (65.7%). Most homes were owned (92.1%) and two-thirds of households reported using septic systems. Approximately 25% of households had at least one person with a college degree in the home.

### 3.1. Perceptions of Water Service Conditions

Among the five conditions, low water pressure was reported the most, with more than 1/3 (35.3%) of participants indicating that they had experienced low water pressure. Intermittent service was the condition reported the least often (14.2%). Water taste, color and/or odor were reported by approximately 20% of all participants, with objectionable tastes being reported with the highest frequency of the three (21.2%). A similar pattern emerged when participants were asked to provide details regarding the frequency of the reported water service conditions; 20% reported low water pressure monthly whereas only 5% reported intermittent service monthly. Monthly reports of taste, odor and color problems were more frequent than other water conditions. For example, only 33% of households that reported intermittent service reported experiencing it monthly, whereas 83% of households that reported objectionable taste reported experiencing that condition monthly.

**Table 1:** Perceptions of water service conditions in a survey of 910 households in rural Alabama.

Reported service conditions (# of households with responses)	Frequency	% reporting the condition (N)	% of those reporting issue that reported it monthly (N/N)
Intermittent service (890)	At Least Once	14.2 (129)	33% (42/129)
	Monthly	4.6 (42)	
Low Water Pressure (887)	At Least Once	35.3 (321)	55% (178/321)
	Monthly	19.6 (178)	
Objectionable Taste (852)	At Least Once	17.0 (155)	83% (128/155)
	Monthly	14.2 (128)	
Objectionable Odor (874)	At Least Once	21.2 (193)	80% (155/193)
	Monthly	17.0 (155)	
Objectionable Color (878)	At Least Once	17.4 (158)	64% (101/158)
	Monthly	11.1 (101)	

### 3.2. Drinking Water Quality 

A summary of drinking water quality measures is presented in Table 2. A total of 855 households had an outside faucet, which permitted the collection of samples for microbial analyses and pressure readings. Average pressure was 462 kPa (67 psi) although there was a substantial range of pressures (from 34 to 1007 kPa, 5–146 psi). Median turbidity was 0.26 NTU and ranged from very low (0.05 NTU) to substantially above Environmental Protection Agency (EPA) standards (13.6 NTU). EPA standards state that for systems that use conventional or direct filtration, turbidity cannot exceed 1 NTU. Median free and total chlorine were 0.7 and 0.9 mg/L, respectively, and very few samples lacked detectable chlorine (3.9% and 1.5%, respectively). Of the households tested, 16.7% and 12.2% of outside and kitchen faucet samples, respectively, were positive for total coliforms. *E. coli* were detected in less than 1% of water samples drawn from kitchen or outside faucets. All water quality parameters, including turbidity, free and total chlorine, pressure and concentration of total coliforms or *E. coli*, were non-normally distributed as determined by Shapiro-Wilk test of normality (*p* < 0.001) and plots.

**Table 2:** Summary of drinking water quality measures for households using small rural drinking water systems in Alabama.

Sample Details	Water Quality Measures					
	Pressure (kPa) **	Turbidity (NTU)	Free Chlorine (mg/L)	Total Chlorine (mg/L)	Outside Total Coliform (MPN/100 mL)	Kitchen Total Coliform (MPN/100 mL)
Number of Observations	855	887	802	802	855	890
Mean	462	0.37	0.90 *	1.1 *	5.7	3.8
Median	427	0.26	0.70 *	0.90 *	<1	<1
Range	34–1000	0.050–14	<0.1–5.9	<0.1–6.2	<1–>200	<1–>200
% Below Detection	NA	NA	3.9%	1.5%	83%	88%

Notes: * 88 observations from one research team member were excluded due to error in measurement for both total and free chlorine; ** 1 kPa ≈ 0.145 psi.

### 3.3. Associations between Perceptions of Water Service Conditions and Measured Drinking Water Quality 

To determine whether or not reported problems surrounding water service delivery were associated with measured water quality, we examined the five reported water service conditions (and their frequency) for associations with the six water quality measures. A summary of these analyses is presented in Table 3 and Table 4. As shown for the analyses in these tables, statistically significant associations were found between reported and measured water quality for two of the five reported water service conditions: intermittent service and low water pressure. 

#### 3.3.1. Associations between Water Delivery Conditions (Intermittent Service and Low Water Pressure) and Measured Drinking Water Quality

As shown in Table 3, a significant difference was detected across frequency of reported intermittent service and free and total chlorine. In this analysis, it was found that as the frequency of reported intermittent service increased, so did the concentration of free and total chlorine (Kruskal-Wallis test *p*-values 0.009 and 0.013 for free and total chlorine, respectively). No other water quality measures were statistically significantly associated with increases in reported intermittent service although some trends were noted. In particular, turbidity increased with increased reporting of intermittent service while pressure decreased, but neither was statistically significant. Presence of total coliform bacteria was variable depending upon the type of sample. For samples that were drawn from an outside faucet, the proportion of samples that were positive for total coliforms decreased with increasing frequency of intermittent service, although this was not a statistically significant association. 

A significant difference was detected across frequency of reported conditions for free chlorine, total chlorine, and gauge-measured water pressure. The group with the most frequently reported low pressure had the highest median values of free and total chlorine concentrations (Kruskal-Wallis *p*-value—0.02). Those that reported low water pressure most frequently had the lowest median values of pressure (Kruskal-Wallis *p*-value—0.003). Household respondents who reported that they never experienced low water pressure had a median pressure of 441 kPa (64 psi), whereas those that reported experiencing low water pressure monthly had a median pressure of 414 kPa (60 psi). In Alabama, all public water systems are required to supply a minimum of 138 kPa (20 psi) of water pressure at the water system meter under normal operating conditions. No maximum pressure guideline exists in Alabama, although the Uniform Plumbing Code requires that static pressure not exceed 552 kPa (80 psi) to avoid damage to plumbing fixtures and equipment [20]. Reports of low water pressure were not statistically associated with turbidity measurements or with the proportion of samples that tested positive for total coliforms, regardless of the household location of the sample collection (kitchen or outside faucet). 

#### 3.3.2. Associations between Aesthetic Characteristics (Taste, Odor, and Color) and Measured Drinking Water Quality

In addition to delivery conditions, we also examined whether aesthetic characteristics of drinking water quality (objectionable taste, odor or color) were associated with measured drinking water quality. Aesthetic conditions, which when reported were mostly reported to occur quite frequently, were not statistically significantly associated with any water quality measures (as shown in Table 4). These aesthetic conditions seemed less sensitive to changes in reported frequency when analyzed for associations with drinking water quality. For example, those that reported never experiencing taste problems had similar median concentrations of free and total chlorine and turbidity as those households who reported experiencing taste problems at least monthly. Likewise, reported odor or color problems were not statistically significantly associated with any water quality parameter. Overall, means and medians fluctuated minimally for these reported conditions. For example, the median free chlorine value for those who never identified odor issues was higher than for those who reported monthly odor issues (0.8 mg/L versus 0.7 mg/L), but this was not a statistically significant association. There were no statistically significant associations with reports of taste and color problems and there did not appear to be a trend with increasing frequency of reporting these problems. 

#### 3.3.3 Examining univariable and multivariable associations using logistic regression models

In our univariable analysis, we examined the independent association between five exposure and six water quality outcome variables (pressure was the only variable treated as an ordinal outcome). The results of the univariable analysis are shown in the Appendix (Table A1). Two of the 30 univariable associations were statistically significant. Of the five reported water delivery and aesthetic conditions, reported objectionable odor was associated with decreased odds of detecting total chlorine in household water samples and reported low water pressure was associated with decreased odds of measured lower water pressure.

**Table 3:** Association between reported intermittent service and low water pressure and measured water quality in a sample of households of three rural counties in Alabama 2012.

Water Delivery Condition	Frequency	Median Free Cl mg/L (N)	Median Total Cl mg/L (N)	Median Turbidity NTU (N)	Median Pressure kPa ^#^ (N)	Kitchen Total Coliform (% positive in 100 mL)	Outside Total Coliform (% positive in 100 mL)
Intermittent Service	Never	0.7 (673)	0.9 (673)	0.26 (741)	441 (715)	12.1%	16.9%
	At Least Once	1.0 (75)	1.2 (75)	0.27 (84)	434 (82)	11.8%	15.9%
	At Least Monthly	1.5 (35)	1.5 (35)	0.34 (42)	469 (39)	16.8%	15.0%
	*p*-Value	0.009 *	0.013 *	0.22 *	0.42 *	0.65 ^†^	0.94 ^†^
Low Water Pressure	Never	0.7 (497)	0.9 (497)	0.27 (549)	441 (528)	12.5%	18.4%
	At Least Once	0.8 (122)	0.9 (122)	0.23 (142)	441 (139)	12.7%	11.6%
	At Least Monthly	0.9 (163)	1.1 (163)	0.27 (174)	414 (167)	10.3%	15.9%
	*p*-Value	0.02 *	0.02 *	0.06 *	0.003 *	0.71†	0.15 ^†^

Notes: * Compared with Kruskal-Wallis test; ^†^ Proportion tested with Pearson Chi-square test; Statistically significant differences in bold; # 1 kPa ≈ 0.145 psi.

**Table 4:** Association between reported taste, odor and color issues and measured water quality in a sample of households of three rural counties in Alabama 2012.

Aesthetic Characteristic	Frequency	Median Free Cl mg/L (N)	Median Total Cl mg/L (N)	Median Turbidity NTU (N)	Median Pressure kPa ^#^ (N)	Kitchen Total Coliform (% positive in 100 mL)	Outside Total Coliform (% positive in 100 mL)
Objectionable Taste	Never	0.8 (578)	0.9 (578)	0.26 (643)	441 (615)	13.0%	17.2%
	At Least Once	0.9 (36)	1.0 (36)	0.23 (38)	414 (37)	2.63%	23.7%
	At Least Monthly	0.8 (140)	0.9 (140)	0.27 (150)	427 (149)	11.9%	12.8%
	*p*-Value	0.37 *	0.58 *	0.57 *	0.70 *	0.16 ^†^	0.22 ^†^
Objectionable Odor	Never	0.8 (628)	0.9 (628)	0.26 (700)	427 (674)	12.2%	16.4%
	At Least Once	0.9 (23)	1.2 (23)	0.24 (27)	482 (25)	11.1%	19.2%
	At Least Monthly	0.7 (119)	0.8 (119)	0.28 (127)	441 (124)	12.5%	16.7%
	*p*-Value	0.69 *	0.42 *	0.53 *	0.61 *	0.98 ^†^	0.89 ^†^
Objectionable Color	Never	0.8 (637)	0.9 (639)	0.27 (703)	427 (677)	11.5%	15.9%
	At Least Once	0.9 (49)	1.0 (49)	0.22 (55)	469 (53)	19.6%	17.0%
	At Least Monthly	0.7 (87)	0.9 (87)	0.24 (99)	441 (99)	13.1%	20.8%
	*p*-Value	0.81 *	0.85 *	0.16 *	0.48 *	0.19†	0.48 ^†^

Notes: * Medians compared with Kruskal-Wallis test; ^†^ Proportion tested with Pearson Chi-square test; # 1 kPa ≈ 0.145 psi.

Due to collinearity of the exposure variables, all multivariable models included one binary exposure variable (intermittent service, low water pressure, objectionable taste, odor or odd color) and three confounding variables—access to sewerage, presence of college graduates in the household and reported race—for each binary water quality measure or for the ordinal water pressure measure. There were no significant interactions between confounders and exposures, therefore only the main effects are reported. To simplify the presentation, all adjusted models are included in Table 5. The ten models that were found to have >10% change in effect when the three confounding variables were included in the model are indicated in the table. The other 20 models did not demonstrate a significant change in effect when the confounding variables were included. Overall, the results of this analysis were similar to the results of the univariable analysis. Two of the 30 associations examined remained statistically significant in the multivariable analysis. Households that reported an odor problem had decreased odds of total chlorine detected in the samples (OR =0.21, 95% CI (0.060–0.77)) and households that reported low water pressure had increased odds (OR =1.40, 95% CI (1.07–1.84)) of measured low water pressure (using the proportional odds model). 

**Table 5:** Multivariable regression examining association between reported delivery and aesthetic conditions, sewerage and measured water quality in a sample of households of three rural counties in Alabama 2012.

Reported delivery and aesthetic conditions	Presence of total coliforms in kitchen samples OR (95%CI)	Presence of total coliforms in outside samples OR (95%CI)	Presence of free chlorine OR (95%CI)	Presence of total chlorine OR (95%CI)	Turbidity > 0.3NTU OR (95%CI)	Log-transformed Pressure Quintiles* OR (95% CI)
Intermittent Service	0.99 (0.55–1.8) ^†^	0.82 (0.47–1.4)^†^	2.2 (0.50–9.4)	0.69 (0.14–3.3) ^†^	1.45 (0.92–2.0)	0.98 (0.66–1.5) ^†, ‡^
Low Water Pressure	0.78 (0.50–1.2) ^†^	0.73 (0.49–1.1)	0.94 (0.44–2.1)	2.5 (0.51–12) ^†^	0.76 (0.57–1.0)	**1.4 (1.1–1.8)**
Objectionable Taste	0.82 (0.48–1.4)	0.80 (0.50–1.3)	0.99 (0.41–2.4)	0.29 (0.080–1.1) ^†^	0.94 (0.67–1.3)	1.1 (0.82–1.5)
Objectionable Odor	1.0 (0.59–1.8)	1.17 (0.66–1.7)	0.92 (0.37–2.3)	**0.21 (0.060–0.77)** ^†^	0.98 (0.68–1.4)	0.94 (0.67–1.3)
Objectionable Color	1.6 (0.95–2.6) ^†^	1.36 (0.78–2.0)	1.5 (0.50–4.4)	0.93 (0.19–4.5) ^†^	0.77 (0.53–1.1)	0.99 (0.70–1.4)

Notes: * Ordinal logistic regression performed on quintiles of log-transformed pressure; ^†^ indicates multivariable model produced ≥10% change in effect size of exposure variable ‡ Logistic regression performed on binary log-pressure when proportional odds assumption was not met; Statistically significant results in bold.

## 4. Discussion

The results of this study were used to evaluate consumers’ perceptions of the delivery and aesthetics of drinking water from small water systems, and how those perceptions were associated with chlorine, turbidity, and microbiological water quality measured at the household. We examined five service conditions: two focused on the level of service delivery, including water pressure and intermittent service, and three focused on the aesthetic conditions of taste, odor and color. Overall, we found few statistically significant associations between these reported conditions and analyzed parameters for drinking water quality. 

### 4.1. Self-Reported Water Delivery Conditions

There is an increasing recognition of the role that drinking water distribution systems may play in infectious disease outbreaks and even endemic disease transmission [21]. It is important for drinking water distribution systems to maintain hydraulic integrity to protect the water from external contaminants. The Committee on Public Water Supply Distribution Systems of the National Research Council defines hydraulic integrity as the capacity to maintain desirable water flow, water pressure, and water age in a distribution system [22]. For this study, we focused on consumer self-reporting of the system’s ability to maintain a desirable water flow and water pressure paired with a single measurement of pressure at each household. 

Our data indicated that 35.3% of participants reported experiencing low water pressure, which is a documented risk factor for outbreaks of waterborne disease [23]. There are many causes of low water pressure events, including main breaks, opening and closing of valves, power failures, flushing of the system, turning pumps on or off, fighting fires, and any other event that creates a sudden change in water pressure. These low-pressure events can last from minutes to hours. Hydraulic integrity relies on the maintenance of adequate water pressure in a distribution system. Loss of water pressure can represent a breach within the system that could result in either backflow (from cross-connections) or contaminant intrusion [24]. 

Intermittent service was reported less frequently than low water pressure; however, 15% of our participants reported experiencing this condition at least once. For our study, intermittent service was defined as an interruption in water service provided to households for any length of time. Interruption in service is associated with infiltration events, stagnation, and potential growth of microbiological contaminants. Distribution systems with intermittent water supply are most vulnerable to intrusion events, and developing countries are more likely to suffer from intermittent water supplies [25]. Under these circumstances, the risk for contamination is high, with several reports of waterborne disease outbreaks and increased rates of gastrointestinal illness linked to intermittent service [26,27]. In economically richer countries, intermittent supply is less common, but these events still occur in locations where infrastructure function is sub-optimal. Previous research conducted in our study area asked households connected to the water supply system about their perceptions of water system performance. Of those participating, 37% of consumers said that they experienced problems with their connection, most commonly intermittent service, while 14% of all participants reported service interruptions as a recurring issue [16].

### 4.2 Prevalence of Self-Reported Aesthetic Conditions

Households in our study reported various concerns about drinking water aesthetics, with almost 20% of households experiencing some problem with taste, odor and/or color. From a previous study conducted in the area, 18% of public water users rated the color of their water as “poor” or “very poor”, and 12% rated the taste of their water as “poor” or “very poor” [16]. Additionally, a study in Norway documented equivalent or higher percentages of households who reported similar concerns (approximately 30% for taste and odor and 15% for rusty color) [28]. A USA-based study determined that the most important reason for consumers to preferentially consume bottled water over tap water was because they did not like the taste/odor of the water [29]. Multiple other studies have demonstrated that perceived aesthetic values such as color, odor, and taste associated with public drinking water supplies are common reasons for seeking alternatives [30,31,32,33,34].

### 4.3. Chlorine Residuals

Maintenance of a disinfectant residual throughout the distribution system helps to preserve the integrity of the system by inactivating microorganisms, indicating system problems, and controlling the growth of biofilms. The EPA-recommended range for free chlorine residual at the household level is 0.3–0.5 mg/L [35]. If free residual chlorine levels are non-detectable at the household level, it is assumed that the water supply is not protected against recontamination. Our data showed the median free chlorine in our samples was 0.7 mg/L, and very few samples (<2%) had no detectable chlorine. The use of chlorine for disinfection of drinking water may produce a bleach-like odor and/or taste, however, the ability of the consumer to detect this residual varies. EPA has not set a standard for these aesthetic effects, but instead established a maximum residual disinfectant level of 4 mg/L for chlorine that is capable of preventing physiological health effects, such as eye and nose irritation and stomach discomfort. Bacterial contamination was infrequent, with 13-16% of both outside and kitchen faucet samples having detectable total coliforms. The results from this study suggested that the measured microbiological water quality from these households was slightly better than what was detected in a previous study in the area. That study reported a higher percentage of samples with no detectable free chlorine, greater than 33% (*N* = 305), and almost 10% of public water system samples were positive for fecal coliforms [16].

### 4.4. Associations between Low Pressure, Intermittent Service and Water Quality

In our initial analysis, higher reported frequencies of low water pressure and intermittent service were associated with increased free and total chlorine but not associated with the presence of total coliforms. It is possible that when the utilities experience these conditions, they attempt to correct the problem by adding additional chlorine. This may be the reason that we saw a decrease in the percentage of samples positive for total coliforms when low water pressure and intermittent service were reported. When we performed univariate and multivariable logistic regression (examining only the presence of chlorine and not the concentration), there was not a statistically significant association between frequency of report of intermittent service or low water pressure and the presence of free or total chlorine in water samples. 

Reported low water pressure was associated with lower measured water pressure. We found this in our initial analysis and also in univariable and multivariable regression analyses. Few studies have examined pressure at the household level, but low pressure could also be associated with increased opportunities for intrusion into pipes. A case study in the United Kingdom found a very strong association between reported low water pressure at the tap and self-reported diarrhea [36]. From these results, the authors concluded that up to 15% of the gastrointestinal illness (GI) reported among the exposed individuals may be associated with burst water mains and pressure loss events, although the study was not specifically designed to test the hypothesis that low water pressure events were associated with self-reported diarrhea. Additionally, a Norwegian epidemiological study indicated that low pressure episodes (defined as incidents where a part of the water distribution network was closed off due to main breaks or maintenance work with presumed loss of water pressure in the distribution system) caused an increased risk of GI illness among water recipients [37]. A study in the USA suggested a mechanism for this increased GI illness by showing that when otherwise satisfactory water distribution pipes experienced a low pressure event, they aspirated enteric organisms that were present in the soil surrounding the pipe [38]. Similarly, a recent study in India reported that households with intermittent service as opposed to continuous supply were more likely to detect *E. coli* and have lower chlorine residual values [5]. Thus, the high prevalence of reported low water pressure and its association with decreased measured pressure in our study group is a cause for concern given this potential for increased disease risk. 

### 4.5. Associations between Aesthetic Conditions and Water Quality

In our preliminary analysis of reported aesthetic conditions and water quality in this setting, we found no statistically significant associations between these and other variables we measured. When we examined the associations using univariable and multivariable techniques, we found a statistically significant association with reported odor problems and decreased odds of detecting total chlorine in the water samples. We emphasize that human perception and perception data are complex, and our measures may not have captured aesthetic characteristics that could be assumed to have a meaningful association with microbial risk. For example, color can result from different types of contaminants (e.g., rust, sediment) that may not correlate well with other measures of water quality. Furthermore, if a household reported poor taste and odor, they often considered it to occur frequently. Perhaps frequently reported taste and odor problems are an indication of general dislike of the water provided by the small water system, and reflect a more general complaint about the quality of service. As a result, the frequency with which these conditions are reported may be less sensitive to actual fluctuations in aesthetic properties of the water. Additionally, aesthetic characteristics are highly subject to variability in what each individual may consider to be undesirable, suggesting that what one individual may find distasteful or aesthetically unpleasing may not bother another person. Further confounding the issue, the threshold for taste and odor varies with the contaminant of concern, adding even more variability. These complexities have been well documented [39]. As a previous study conducted in the region did not find associations between consumer reported intensity of water condition and water contamination [16], this study focused on overall perceptions (*i.e.*, non-scaled responses).

Previous studies have suggested that aesthetic characteristics are associated with user perception of risk [10,28]. A study that surveyed residents of two community water systems determined that water contamination problems contributed to increased levels of risk perception from those individuals drinking tap water [29]. They also suggested that the level of awareness of the problem affected risk perception [29]. When the utility was unable to correct the condition in a timely manner, the consumer’s perception of risk increased [29]. Limited to a single sample at each household and looking into general perception measurements, our results suggest limited association between perceptions and objectively measurable factors that are known to be associated with microbial risk in this setting. Our approach limits the ability to provide exact links to these perceptions but our intention was to provide a more general sense of how the consumers felt about the water and evaluate the water quality at a single point in time. Furthermore, water quality measures were taken only once for each household and single samples are insufficient as indicators of long-term water quality in water supplies.

## 5. Conclusions 

Data from this study support the following primary conclusions: (1) self-reported aesthetic data from consumers were found to have no or limited associations with measured water quality parameters that are known to be associated with microbial risk (pressure, turbidity, chlorine residuals, total coliforms); (2) self-reported pressure data were associated with measured pressure at the household level, indicating that self-reported data on this parameter may be reliable; and (3) reported low water pressure and intermittent service were associated with elevated free and total chlorine concentrations measured at the point of sampling but had limited association with presence or absence of these residuals.

## Supplementary Files

Supplementary File 1 Supplementary Information (DOCX, 139 KB)

## Appendix

**Table A1:** Univariable associations between reported delivery and aesthetic conditions and measured water quality in a sample of households of three rural counties in Alabama 2012.

Reported delivery and aesthetic conditions	Presence of total coliforms in kitchen samples OR (95%CI)	Presence of total coliforms in outside samples OR (95%CI)	Presence of free chlorine OR (95%CI)	Presence of total chlorine OR (95%CI)	Turbidity > 0.3 NTU OR (95%CI)	Log-transformed Pressure Quintiles * OR (95% CI)
Intermittent Service	1.12 (0.64–1.95)	0.92 (0.54–1.56)	2.25 (0.53–9.63)	0.81 (0.17–3.76)	1.36 (0.93–1.99)	0.89 (0.59–1.34) ^‡^
Low Water Pressure	0.89 (0.58–1.37)	0.71 (0.49–1.06)	1.04 (0.49–2.21)	2.94 (0.63–13.34)	0.78 (0.59–1.04)	**1.43 (1.10–1.85)**
Objectionable Taste	0.75 (0.44–1.26)	0.85 (0.54–1.34)	1.00 (0.42–2.37)	0.42 (0.13–1.33)	0.98 (0.70–1.36)	1.11 (0.82–1.49)
Objectionable Odor	1.00 (0.59–1.70)	1.11 (0.70–1.76)	0.94 (0.38–2.33)	**0.31 (0.10–0.99)**	1.02 (0.72–1.46)	0.95 (0.68–1.32)
Objectionable Color	1.41 (0.86–2.31)	1.27 (0.81–2.01)	1.46 (0.50–4.24)	1.07 (0.23–4.93)	0.78 (0.54–1.12)	0.97 (0.70–1.35)

Note: ***** Ordinal logistic regression performed on quintiles of log-transformed pressure. Odds ratios derived from modeling the probability of being in a lower level of pressure are reported; ^‡^ Logistic regression performed on binary pressure when proportional odds assumption was not met; Statistically significant results in bold.
